# Are Guidelines Needed for the Diagnosis and Management of Incipient Alzheimer's Disease and Mild Cognitive Impairment?

**DOI:** 10.4061/2010/417615

**Published:** 2010-08-17

**Authors:** Katie Palmer, Massimo Musicco, Carlo Caltagirone

**Affiliations:** ^1^Fondazione Santa Lucia, Istituto di Ricovero e Cura a Carattere Scientifico (IRCCS), Rome 00179, Italy; ^2^Institute of Biomedical Technologies, CNR, 20090, Segrate, Italy; ^3^Department of Neuroscience, University of Rome Tor Vergata, Rome 00173, Italy

## Abstract

Current research is aiming to push the boundaries of the point at which a diagnosis of Alzheimer Disease (AD) can be made. Clinical syndromes such as Mild Cognitive Impairment (MCI) and various clinical and biological markers of AD may help to identify people in the early stage of AD, before a full dementia syndrome is present. In the first part of this paper, we discuss whether MCI represents incipient AD, and examine some of the methods currently used in research to identify AD patients in the preclinical phase. In the second part, we discuss whether specific guidelines are needed for the diagnosis and management of MCI and incipient AD, and consider the potential impact of this on clinical practice and public health from the perspective of patients, caregivers, and healthcare providers.

## 1. Introduction: The Concept of MCI and Incipient AD

Currently, dementia of the Alzheimer type is diagnosed clinically according to diagnostic criteria [1]. These criteria include deficits in memory and other cognitive functions, and the symptoms have a gradual onset and progressive deterioration. The H. Braak and E. Braak [2, 3] staging describes an increasing burden of the neuropathological hallmarks of Alzheimer disease (AD), neurofibrillary tangles, and amyloid plaques, with increasing dementia severity, mainly starting in the brain regions associated with memory functioning such as the hippocampus and entorhinal cortex. Evidence now clearly shows that the disease starts before the symptoms of a full dementia syndrome are present. AD related pathology, such as beta amyloid deposition, may already begin occurring even ten to twenty years before a clinical diagnosis of dementia can be made [4]. Furthermore, deficits in cognitive functioning may start to appear three [5], six, or even up to ten years [6] before a clinical diagnosis of AD. 

Several syndromes that identify patients at high risk of developing dementia—or “preclinical, early AD” syndromes—have been proposed, including Mild Cognitive Impairment (MCI) and others; for a review see Mariani and colleagues [7]. A decade ago, Petersen et al. [8] proposed diagnostic criteria to describe patients who were neither normal nor demented, but had a mild form of cognitive impairment characterized by memory deficits. The MCI paradigm is based on the assumption that patients with AD develop symptoms, particularly memory impairment, gradually and, therefore, there may be an intermittent stage between normal aging and dementia characterized primarily by episodic memory deficits. These MCI patients have a high risk of progressing to a full dementia syndrome within a few years [8], although, as discussed later, the evolution is heterogeneous. 

In the last decade, much focus has been paid to identifying clinically diagnosable syndromes of incipient AD. Criteria for MCI, and other syndromes, have been revised several times [9–11], with interest in identifying specific characteristics of MCI patients who are at the highest risk of progressing to AD. People with MCI progress to dementia at a rate of about 6% to 15% per year [7, 8, 12, 13], although the condition is heterogeneous and some MCI patients do not develop dementia. As discussed in a recent systematic review of MCI [7], a number of factors have been suggested to further predict conversion to AD in MCI patients, and it is becoming increasingly evident that biomarkers, such as cerebrospinal fluid (CSF) markers including beta-amyloid_1-42_ and total tau protein, may have a good accuracy for identifying impending AD in MCI patients [14] compared to healthy controls. Although the differential diagnostic capacity of using CSF markers between the preliminary states of various dementia disorders has not yet been thoroughly studied, the discoveries regarding AD are pushing the boundaries as to when AD can be identified and diagnosed in individuals.

## 2. Guidelines for AD and Dementia

Guidelines for the diagnosis and management of AD and other dementias are regularly published, updated, and revised in the USA and elsewhere in the world [15–19]. The latest European recommendations for the diagnosis and management of AD were published by the European Federation of Neurological Societies (EFNS) [17]. These evidence-based guidelines were created through a consensus process to provide guidance for physicians concerning diagnostic evaluation and management of dementia, including pharmacological and nonpharmacological treatment, management of behavioral and psychological symptoms of dementia (BPSD), counseling and support for caregivers, and so forth. The EFNS guidelines state that the recommendations are relevant only to dementia, and do not include MCI. Recently, Dubois et al. [20] published new diagnostic criteria for the early detection of AD. These were proposed as research criteria, rather than for clinical diagnosis and patient management, and incorporate clinical evidence of episodic memory loss (isolated or with other cognitive deficits) with biomarkers (CSF, neuroimaging, genetic) to identify patients in an early, predementia stage of AD. The criteria are novel because rather than focusing on the dementia syndrome they focus on clinical, biological, structural, and biochemical presence of AD in a predementia phase. These, and other, criteria for syndromes of early preclinical phases of AD raise controversial questions in the scientific and medical world. Should physicians diagnose MCI or incipient AD? Should MCI patients be treated? How should physicians and healthcare workers manage the healthcare of patients with MCI? Should dementia guidelines be revised to incorporate MCI? In this paper, we will discuss the disadvantages and advantages of omitting MCI and incipient AD from dementia guidelines, and discuss the potential impact on clinical practice and public health from the perspective of patients, caregivers, and healthcare providers.

## 3. Does MCI Represent Incipient AD?

The first relevant question to ask in this discussion is whether syndromes such as MCI truly represent an early preclinical phase of AD. Research both from clinical- and population-based samples suggest that MCI patients have a high risk of progressing to a full dementia syndrome; a meta-analyses [12] of longitudinal studies of individuals with MCI reported an annual conversion rate of 8.1% in clinical settings, and 6.8% in community-based settings. Pooled-analysis [13] suggests that about one third of MCI patients will convert to AD, with a higher conversion rate in the first few years following MCI diagnosis. However, it has consistently been shown that not all MCI patients will develop AD [7, 12], and that cognitive impairment in the elderly has heterogeneous risk factors [21] and potentially diverse etiologies and causes [11]. Although neuropathological studies on MCI patients indicate that pathological findings in the brain structures involved in memory represent a transition between normal aging and AD, many concurrent pathological abnormalities are also present in these patients, including vascular lesions and argyrophilic grain disease [22]. Further, neuropathological investigations in patients who progressed from MCI to AD show a heterogeneous pathological outcome [23]. In addition, not all AD patients pass through a clinically identifiable stage of MCI with subjective and objective memory deficits present prior to dementia diagnosis [24]. Thus, the evidence suggests that, although MCI does represent a group of individuals with a high risk of progressing to AD, MCI does not equal incipient AD because not all MCI patients will develop AD. 

As mentioned previously, much work has been done to further characterize MCI patients to identify which individuals are most likely to develop AD. Therefore, although patients with a clinical diagnosis of MCI do not necessarily have AD, there are subsets of MCI patients who have a neurodegenerative AD etiology, and are in the early phase of AD. Functional markers, cognitive testing, hippocampal and entorhinal cortex atrophy, neuropsychiatric disorders, genetic factors such as Apolipoprotein E (ApoE) epsilon 4 (*ε*4) allele, increased age, low education, and motor dysfunction are all markers that have been shown to identify which MCI patients have the highest risk of developing AD [7]. It is also evident that combining factors (including neuroimaging, clinical, and sociodemographic markers) [25, 26] increases the prediction for identifying which MCI patients will progress to AD. Indeed, Dubois and colleagues' research criteria for the early detection of AD [20] incorporate clinical evidence of cognitive deficits with neuroimaging, CSF, and genetic biomarkers. However, it is not yet clear which markers and cutoff values are the best for identifying incipient AD patients; although Dubois and colleagues make some suggestions for biomarkers, such as hippocampal volume loss, it was emphasized that research should identify and validate other biomarkers and neuroimaging regions of interest in the future. It is worth noting that although combining markers will help to increase the positive predictive value for identifying which MCI patients will develop AD, ultimately this may lead to a drop in the other diagnostic values. Tests and markers with high positive predictive value are likely to have lower sensitivity, especially at the population level. Therefore, very few patients with AD will be able to be identified in the early stage of the disease.

As discussed elsewhere in this special issue, much research effort has been made in the field of biomarkers, particularly CSF, for identifying patients in the early, predementia, phase of AD. A multicenter study of the CSF markers in MCI patients has shown innovative results concerning the use of beta-amyloid_1-42_, total tau protein, and tau phosphorylated at position threonine 181, which was found to have a good accuracy for identifying impending AD in MCI patients [14]. This was one of the first studies to pool the data from a large range of single center studies. It showed that the positive predictive value of these CSF markers for identifying incipient AD in MCI patients was 62%, with a sensitivity of 83%. However, despite these promising results, the findings should be viewed with caution. An editorial by Petersen and Tojanowski [27] highlighted the clinical variability of the MCI patients as well as the variability in CSF measurements in the different centers in Mattsson and colleagues' work. Further, they acknowledged that although the sensitivity and specificity of the CSF markers were adequate for screening, they did not have high enough accuracy to be used as diagnostic tests, although they may be useful for use in clinical trials [27]. Interestingly, the positive predictive value showed that 62% of MCI patients developed AD when a combination of the three CSF markers was used [14]. However, this positive predictive value needs to be compared with other markers and clinical syndromes. Before taking into consideration other markers, the cumulative conversion rate from MCI to AD across studies is 31.4% [13]. Furthermore, other studies have shown that this positive predictive value can be increased by combining other, less invasive, markers. For example, a prediction model using five factors (functional and olfactory markers, cognitive testing, and MRI measures of hippocampal volume and entorhinal cortex volume) had a positive predictive value of more than 80% for identifying incipient AD in MCI patients [25].

There are important limitations to consider when examining clinical and laboratory markers in MCI patients. First, laboratory samples are mainly taken from clinical patient populations, which may not be representative of the general population. Consequently, the sensitivity of a laboratory measure evaluated in a clinical sample does not reflect the level of sensitivity of the test if used at the population level. Second, a study in the general population showed that half of AD patients have no subjective complaints about their memory performance up to one to three years before AD diagnosis, and one fifth show absolutely no cognitive deficits [24]. This highlights the heterogeneity of patients in the preclinical phase of AD. It is likely that patients who seek medical advice for cognitive problems are at a more advanced stage of cognitive impairment, closer to the diagnosis of AD, which will affect the accuracy of measurements of sensitivity and predictive values in markers such as CSF values. Finally, the multicenter study by Mattsson et al. [14] found that the diagnostic validity of CSF markers had less accuracy in the multicenter analysis than the accuracy reported in single-center settings. This highlights the importance of replicating positive results from single centers, which are often conducted on small study populations, and the need for more multicenter trials and meta-analyses to determine the accuracy of biomarkers for identifying incipient AD.

## 4. Do We Need Guidelines for the Diagnosis and Management of MCI?

MCI is a frequently occurring syndrome in the elderly. A European longitudinal study [28] reported an incidence rate per 1000 person years of between 11.4 for amnestic-MCI and 33.8 for other types of cognitive impairment, and prevalence data suggests that cognitive impairment syndromes in younger nondemented elderly actually exceed dementia prevalence [29]. Given that MCI is a frequent syndrome in older people, and that individuals with MCI have a high risk of progressing to AD [7, 12], this raises the question of what should be done with such patients. As mentioned earlier, guidelines for the management of dementia often do not include recommendations for patients with MCI. However, many of the issues regarding both diagnosis and management of dementia may also be relevant to the large number of elderly persons suffering from MCI, who may or may not be in the early stage of AD. Clinically, MCI could be viewed, not as a disease or disorder per se, but as a risk factor for AD. Risk factors such as this are commonly addressed in other areas of medicine. Many conditions have been shown to predispose an individual to a higher risk of developing another medical condition, such as hypertension for stroke and myocardial infarction, dyslipidemia for stroke and other vascular disease, diabetes for osteoporosis and so forth. In good clinical practice, clinicians explain the risks associated with such conditions with their patients, and plan appropriate care and management. In this way, MCI should be viewed as a clinical syndrome that has a high risk of progressing to AD. Thus, if clinicians were to consider MCI, not as incipient AD, but as a risk factor for developing AD, certain issues concerning management and treatment that have been raised for dementia, may be relevant also to MCI.

## 5. Treatment of MCI

First, although dementia disorders such as AD are currently not curable, there are a number of symptomatic pharmaceutical treatments that are recommended in guidelines for dementia [17, 30]. Conversely, there are currently no approved drugs for the treatment of MCI or early preclinical AD. Clinical trials on MCI patients have had limited success; none of the trials, including donepezil, rivastigmine, rofecoxib, galantamine, and vitamin E, have demonstrated convincing effects for delaying progression from MCI to AD [31, 32]. However, other trials are ongoing, and data on subsets of MCI patients have identified potentially promising avenues for future research. For example, although randomized-control trials on cholinesterase inhibitors such as donepezil have shown little or no reduction in the risk of conversion from MCI to AD [33–35], the results of one trial suggested that MCI patients with depressive symptoms treated with donepezil had a lower risk of conversion to AD [36]. Without the availability of effective treatment strategies, and in the absence of approved drugs for the treatment of MCI or incipient AD, one may question the benefits of diagnosing such syndromes. Thus, dementia guidelines, such as those proposed by EFNS, might be right to avoid discussing disease-specific treatment strategies for MCI, since MCI per se is not a disease, and currently there are no approved drugs for MCI. However, pharmacological treatments are not the only recommendations suggested in current guidelines for dementia, and the other recommendations might be of relevance to MCI patients as well as those with dementia, including suggestions for diagnosis, management of BPSDs, and support for families and caregivers.

## 6. Nonpharmacological Treatment and Interventions

Although there are no currently approved pharmacological treatments for MCI, nonpharmacological interventions have also been the focus of recent research. Management of risk factors and somatic disorders, lifestyle changes, and cognitive intervention programmes may be beneficial to certain patients, and may be advantageous as they might have less side effects and risks than pharmacological drugs. Cognitive rehabilitation studies in MCI [37–39] have showed improvement in ADL functioning, mood, and memory following training, although there is no evidence for delayed progression from MCI to AD. Further, if MCI patients are told of their high risk of developing AD, there are possibilities for modifying risk factors associated with AD, such as diet, somatic, and lifestyle factors [40]. Further, as it has been shown that MCI has heterogeneous risk factors [21] and that MCI may have diverse somatic, psychiatric, and neurological etiologies [11], the modification of somatic and lifestyle factors may be beneficial to the overall health of MCI patients, even those who are not in a preclinical phase of AD.

## 7. Disclosure of MCI Diagnosis

There is ongoing debate as to whether a formal diagnosis of MCI should be made and disclosed to patients in the clinical setting. The fact is that increasing numbers of elderly people are approaching physicians complaining of memory problems, and there is a high incidence of both MCI and other syndromes of cognitive impairment in the elderly [28]. There may be some benefits to disclosing a diagnosis of MCI to a patient and discussing the associated risk of developing AD. First, it allows patients to plan for their future before their cognitive impairment becomes severe, including financial planning, and the planning of care and living arrangements for the future. Second, in some cases it may be helpful for individuals to have a formal diagnosis that somewhat explains their symptoms. From the physician's perspective, giving an early diagnosis may be beneficial as the patient will have better cognitive capacities to understand the diagnosis and discuss future treatment and care options; indeed, studies have shown that physicians are often reluctant to disclose a diagnosis of dementia in more severe stages of the disease due to lack of comprehension by the patient [41]. Furthermore, physicians may be concerned that disclosing a diagnosis of dementia may trigger catastrophic events in the patient such as depression or suicide. However, a study by Carpenter et al. [42] examined changes in depression and anxiety both in patients and their relatives before and after the diagnosis of dementia. They found that there were no changes in depression status in either patients or their next-of-kin after a disclosure of a dementia diagnosis, but levels of anxiety decreased substantially after receiving the diagnosis. This supports the idea that receiving a formal diagnosis that explains the symptoms and memory loss can actually be beneficial to a patient. 

Interesting lessons can also be learned from studies investigating the effect of disclosing genetic risk of AD to individuals, such as ApoE *ε*4, a cardiovascular risk factor which is also associated with an increased risk of developing AD [43]. Both MCI and the ApoE *ε*4 genotype are associated with a higher risk of AD but do not represent a definite risk; that is, not all patients with MCI or ApoE *ε*4 will develop AD. The REVAL study group [44] investigated the effect of disclosing AD risk (both ApoE genotype and a numeric risk estimate) to family members of AD patients using a randomized-control trial design. They found no differences in short-term psychological symptoms such as anxiety, depression, or distress between people who were told that they had a genetic risk of developing AD (ApoE *ε*4 carriers) than those who were not told their genotype. However, when questioned after one year, individuals were more likely to recall their genotype than their numerical lifetime risk estimate of AD [45]. The authors suggested that clinicians, therefore, needed to find an appropriate manner to communicate AD risk and probabilities. Most interestingly, however, was that disclosure of genetic AD risk was associated with a change in behaviors; a large proportion of individuals made changes to their long-term healthcare insurance after learning they were ApoE *ε*4 carriers [46] and more than half made changes to their health behavior, such as vitamin intake and exercise [47]. Although specific studies on the disclosure of MCI diagnosis are needed, the results of there studies on disclosing genetic AD risk suggest potentially beneficial effects of informing an at-risk individual that they have a high risk of developing AD.

## 8. Recommendations for the Management of BPSD

Although there are no approved treatments for MCI, patients with this syndrome might have other related symptoms that can be treated. The EFNS guidelines for dementia stipulate that physicians should be aware of the importance of treating behavioral and psychiatric symptoms for the potential benefit of the patients and carers. This may also be relevant to MCI patients. Studies both from clinical- and population-based samples consistently report a high burden of BPSD in MCI patients, particularly depression, anxiety, and irritability (for a review see Monastero and colleagues [48]). Indeed the pattern of BPSD in MCI are similar to those observed in patients with dementia, and symptoms have been observed in more than one third of patients, with many studies reporting a higher prevalence [48], even up to 85%. Nevertheless, the role of BPSD in MCI remains unclear and, thus, treatment of specific symptoms or psychiatric disorders in this group of patients is controversial. A population-based study showed that anxiety in MCI patients was associated with an ongoing AD related neurodegeneration [49], and clinical-based studies suggest a similar role for apathy [50–52]. In contrast, depression in MCI is generally not associated with progression to AD [49, 53]. There are currently no clinical trials investigating the effect of treating BPSD and psychiatric disorders in MCI patients, and thus it is not known whether pharmacological treatments are effective either in slowing the progression from MCI to AD or in reducing the severity of neuropsychiatric symptoms in these patients. Indeed, the situation is similar for patients already in the stage of clinical dementia; current guidelines for dementia [17] highlight these controversies.

## 9. Patient Guidelines and Advice

Guidelines on MCI for healthcare workers as well as patients have already been published in America. In 2010, the American Academy of Physician Assistants [54] published guidelines for physician assistants, stating that they should be able to recognize and treat MCI and dementia. Specific suggestions were made for screening tools, cognitive and somatic evaluation of at-risk patients, as well as tentative recommendations for treatment strategies. A Patient Page, published in the Journal of the American Medical Association [55], aims to provide patients and families with general information about MCI, including possible conditions associated with MCI, a description of diagnostic techniques, and some recommendations for prevention, mainly the management of high blood pressure and other chronic conditions, as well as social activities and dietary changes. A patient and family fact sheet on MCI, published in The Neurologist [56] describes potential causes of cognitive impairment in the elderly, rather than focusing specifically on AD, and describes diagnostic procedures and examination that might be conducted on patient with suspected cognitive problems. It also makes suggestions for life style changes, including social and dietary changes.

## 10. Conclusions

In conclusion, MCI is not a disease or disorder, but a risk factor for developing dementia and AD. Although studies examining biological markers and other factors in MCI patients have identified markers that further increase the risk of developing AD in these patients, we are still not at a stage where we can accurately identify with 100% accuracy which patients are in the early phase of AD. Consequently, current guidelines for the diagnosis and management of AD and dementia often do not include specific suggestions for MCI. Despite this, many of the recommendations made for patients with dementia, may also be relevant to people with MCI. Therefore, peer-reviewed, consensus guidelines for the diagnosis and management of incipient AD or MCI might be beneficial in the future. However, it is imperative to focus future research on identifying more accurate diagnostic markers to define incipient AD, and to study the effect of informing patients that they have a high risk of developing AD, in terms of psychological reactions and potential benefits for planning appropriate management and prevention strategies in MCI patients.

## References

[1] McKhann G, Drachman D, Folstein M, Katzman R, Price D, Stadlan EM (1984). Clinical diagnosis of Alzheimer’s disease: report of the NINCDS-ADRDA work group under the auspices of Department of Health and Human Services Task Force on Alzheimer’s disease. *Neurology*.

[2] Braak H, Braak E (1991). Neuropathological stageing of Alzheimer-related changes. *Acta Neuropathologica*.

[3] Braak H, Braak E (1995). Staging of Alzheimer’s disease-related neurofibrillary changes. *Neurobiology of Aging*.

[4] Jack CR, Lowe VJ, Weigand SD (2009). Serial PIB and MRI in normal, mild cognitive impairment and Alzheimers disease: implications for sequence of pathological events in Alzheimers disease. *Brain*.

[5] Bäckman L, Jones S, Berger A-K, Laukka EJ, Small BJ (2005). Cognitive impairment in preclinical Alzheimer’s disease: a meta-analysis. *Neuropsychology*.

[6] Amieva H, Le Goff M, Millet X (2008). Prodromal Alzheimer’s disease: successive emergence of the clinical symptoms. *Annals of Neurology*.

[7] Mariani E, Monastero R, Mecocci P (2007). Mild cognitive impairment: a systematic review. *Journal of Alzheimer’s Disease*.

[8] Petersen RC, Smith GE, Waring SC, Ivnik RJ, Tangalos EG, Kokmen E (1999). Mild cognitive impairment: clinical characterization and outcome. *Archives of Neurology*.

[9] Petersen RC, Doody R, Kurz A (2001). Current concepts in mild cognitive impairment. *Archives of Neurology*.

[10] Petersen RC (2004). Mild cognitive impairment as a diagnostic entity. *Journal of Internal Medicine*.

[11] Winblad B, Palmer K, Kivipelto M (2004). Mild cognitive impairment—beyond controversies, towards a consensus: report of the International Working Group on Mild Cognitive Impairment. *Journal of Internal Medicine*.

[12] Mitchell AJ, Shiri-Feshki M (2009). Rate of progression of mild cognitive impairment to dementia—meta-analysis of 41 robust inception cohort studies. *Acta Psychiatrica Scandinavica*.

[13] Mitchell AJ, Shiri-Feshki M (2008). Temporal trends in the long term risk of progression of mild cognitive impairment: a pooled analysis. *Journal of Neurology, Neurosurgery and Psychiatry*.

[14] Mattsson N, Zetterberg H, Hansson O (2009). CSF biomarkers and incipient Alzheimer disease in patients with mild cognitive impairment. *Journal of the American Medical Association*.

[15] Alzheimer’s Association (2009). 2009 Alzheimer’s disease facts and figures. *Alzheimer’s and Dementia*.

[16] Kalaria RN, Maestre GE, Arizaga R (2008). Alzheimer’s disease and vascular dementia in developing countries: prevalence, management, and risk factors. *The Lancet Neurology*.

[17] Waldemar G, Dubois B, Emre M (2007). Recommendations for the diagnosis and management of Alzheimer’s disease and other disorders associated with dementia: EFNS guideline. *European Journal of Neurology*.

[18] Caltagirone C, Bianchetti A, Di Luca M (2005). Guidelines for the treatment of Alzheimer’s disease from the Italian association of psychogeriatrics. *Drugs and Aging*.

[19] Musico M, Caltagirone C, Sorbi S (2004). Italian Neurological Society guidelines for the diagnosis of dementia: revision I. *Neurological Sciences*.

[20] Dubois B, Picard G, Sarazin M (2009). Early detection of Alzheimer’s disease: new diagnostic criteria. *Dialogues in Clinical Neuroscience*.

[21] Monastero R, Palmer K, Chengxuan Q, Winblad B, Fratiglioni L (2007). Heterogeneity in risk factors for cognitive impairment in non-demented elderly: a population based longitudinal study. *American Journal of Geriatric Psychiatry*.

[22] Petersen RC, Parisi JE, Dickson DW (2006). Neuropathologic features of amnestic mild cognitive impairment. *Archives of Neurology*.

[23] Jicha GA, Parisi JE, Dickson DW (2006). Neuropathologic outcome of mild cognitive impairment following progression to clinical dementia. *Archives of Neurology*.

[24] Palmer K, Bäckman L, Winblad B, Fratiglioni L (2008). Early symptoms and signs of cognitive deficits might not always be detectable in persons who develop Alzheimer’s disease. *International Psychogeriatrics*.

[25] Devanand DP, Liu X, Tabert MH (2008). Combining early markers strongly predicts conversion from mild cognitive impairment to Alzheimer’s disease. *Biological Psychiatry*.

[26] Kantarci K, Weigand SD, Przybelski SA (2009). Risk of dementia in MCI: combined effect of cerebrovascular disease, volumetric MRI, and 1H MRS. *Neurology*.

[27] Petersen RC, Trojanowski JQ (2009). Use of Alzheimer disease biomarkers: potentially yes for clinical trials but not yet for clinical practice. *Journal of the American Medical Association*.

[28] Caracciolo B, Palmer K, Monastero R, Winblad B, Bäckman L, Fratiglioni L (2008). Occurrence of cognitive impairment and dementia in the community: a 9-year-long prospective study. *Neurology*.

[29] De Ronchi D, Berardi D, Menchetti M (2005). Occurrence of cognitive impairment and dementia after the age of 60: a population-based study from northern Italy. *Dementia and Geriatric Cognitive Disorders*.

[30] Neugroschl J, Sano M (2009). An update on treatment and prevention strategies for Alzheimer’s disease. *Current Neurology and Neuroscience Reports*.

[31] Jelic V, Kivipelto M, Winblad B (2006). Clinical trials in mild cognitive impairment: lessons for the future. *Journal of Neurology, Neurosurgery and Psychiatry*.

[32] Farlow MR (2009). Treatment of mild cognitive impairment (MCI). *Current Alzheimer Research*.

[33] Petersen RC, Thomas RG, Grundman M (2005). Vitamin E and donepezil for the treatment of mild cognitive impairment. *The New England Journal of Medicine*.

[34] Sobów T, Kłoszewska I (2007). Cholinesterase inhibitors in mild cognitive impairment: a meta-analysis of randomized controlled trials. *Neurologia i Neurochirurgia Polska*.

[35] Doody RS, Ferris SH, Salloway S (2009). Donepezil treatment of patients with MCI: a 48-week randomized, placebo-controlled trial. *Neurology*.

[36] Lu PH, Edland SD, Teng E, Tingus K, Petersen RC, Cummings JL (2009). Donepezil delays progression to AD in MCI subjects with depressive symptoms. *Neurology*.

[37] Jean L, Bergeron M-E, Thivierge S, Simard M (2010). Cognitive intervention programs for individuals with mild cognitive impairment: systematic review of the literature. *American Journal of Geriatric Psychiatry*.

[38] Talassi E, Guerreschi M, Feriani M, Fedi V, Bianchetti A, Trabucchi M (2007). Effectiveness of a cognitive rehabilitation program in mild dementia (MD) and mild cognitive impairment (MCI): a case control study. *Archives of Gerontology and Geriatrics*.

[39] Kurz A, Pohl C, Ramsenthaler M, Sorg C (2009). Cognitive rehabilitation in patients with mild cognitive impairment. *International Journal of Geriatric Psychiatry*.

[40] Middleton LE, Yaffe K (2009). Promising strategies for the prevention of dementia. *Archives of Neurology*.

[41] Johnson H, Bouman WP, Pinner G (2000). On telling the truth in Alzheimer’s disease: a pilot study of current practice and attitudes. *International Psychogeriatrics*.

[42] Carpenter BD, Xiong C, Porensky EK (2008). Reaction to a dementia diagnosis in individuals with Alzheimer’s disease and mild cognitive impairment. *Journal of the American Geriatrics Society*.

[43] Purnell C, Gao S, Callahan CM, Hendrie HC (2009). Cardiovascular risk factors and incident alzheimer disease: a systematic review of the literature. *Alzheimer Disease and Associated Disorders*.

[44] Green RC, Roberts JS, Cupples LA (2009). Disclosure of APOE genotype for risk of Alzheimer’s disease. *The New England Journal of Medicine*.

[45] Eckert SL, Katzen H, Roberts JS (2006). Recall of disclosed apolipoprotein E genotype and lifetime risk estimate for Alzheimer’s disease: the REVEAL study. *Genetics in Medicine*.

[46] Zick CD, Mathews CJ, Roberts JS, Cook-Deegan R, Pokorski RJ, Green RC (2005). Genetic testing for Alzheimer’s disease and its impact on insurance purchasing behavior. *Health Affairs*.

[47] Chao S, Roberts JS, Marteau TM, Silliman R, Cupples LA, Green RC (2008). Health behavior changes after genetic risk assessment for Alzheimer disease: the REVEAL study. *Alzheimer Disease and Associated Disorders*.

[48] Monastero R, Mangialasche F, Camarda C, Ercolani S, Camarda R (2009). A systematic review of neuropsychiatric symptoms in mild cognitive impairment. *Journal of Alzheimer’s Disease*.

[49] Palmer K, Berger AK, Monastero R, Winblad B, Bäckman L, Fratiglioni L (2007). Predictors of progression from mild cognitive impairment to Alzheimer disease. *Neurology*.

[50] Vicini Chilovi B, Conti M, Zanetti M, Mazzù I, Rozzini L, Padovani A (2009). Differential impact of apathy and depression in the development of dementia in mild cognitive impairment patients. *Dementia and Geriatric Cognitive Disorders*.

[51] Robert PH, Berr C, Volteau M (2006). Apathy in patients with mild cognitive impairment and the risk of developing dementia of Alzheimer’s disease. A one-year follow-up study. *Clinical Neurology and Neurosurgery*.

[52] Palmer K, Di Iulio F, Varsi A (2010). Neuropsychiatric predictors of progression from amnestic-Mild Cognitive Impairment to Alzheimer’s Disease. The role of depression and apathy. *Journal of Alzheimer’s Disease*.

[53] Rozzini L, Chilovi BV, Trabucchi M, Padovani A, Modrego PJ (2005). Depression is unrelated to conversion to dementia in patients with mild cognitive impairment. *Archives of Neurology*.

[54] Boissonneault GA (2010). MCI and dementia: diagnosis and treatment. *Journal of the American Academy of Physicians Assistants*.

[55] Torpy JM, Lynm C, Glass RM (2009). JAMA patient page. Mild cognitive impairment. *Journal of the American Medical Association*.

[56] Kernich CA (2009). Patient and family fact sheet. Mild cognitive impairment. *Neurologist*.

